# A LC-MS-Based Method for Quantification of Biomarkers from Serum of Allergic Rats

**DOI:** 10.3390/molecules15053356

**Published:** 2010-05-07

**Authors:** Yu Jing Bai, Xiao Yan Gao, Jian Qiu Lu, Hong Gui Zhang

**Affiliations:** 1 School of Chinese Materia Medica, Beijing University of Chinese Medicine, No.6, Zhonghuan South Road, Wangjing, Chaoyang district, Beijing 100102, China; E-Mail: jingjing8371332@sina.com (Y.J.B); 2 Science and Technology Development Center for Traditional Chinese Medicine in Beijjing University of Chinese Medicine, No.11, East 3rd Ring Road, Chaoyang District, Beijing, 100029,China; E-Mail: lingzhiwu_2008@163.com (X.Y.G)

**Keywords:** LC-MS/MS, biomarker, serum, allergic rats

## Abstract

Allergies are highly complex disorders with clinical manifestations ranging from mild oral, gastrointestinal, recurrent wheezing, and cutaneous symptoms to life-threatening systemic conditions. The levels of arachidonic acid, eicosanoids, histamine, organic acids and valine are considered to have a variety of physiological functions in connection with allergies. In this research, we have developed a RP-LC/MS method to separate and quantitate six different potential endogenous biomarkers, including leukotrieneB_4 _(LTB_4_), prostaglandinD_2 _(PGD_2_), arachidonic acid (AA), histamine (HI), lactic acid (LA) and valine (VAL), from serum of rats with ovalbumin (OVA)-induced allergy and normal rats, and the discrepancies between the model group and the control group were compared. The separation was performed on a Prevail C_18 _column (250 mm × 4.6 mm, 5 μm) with a gradient elution of acetonitrile with 0.1% formic acid (v/v) and 10 mM ammonium formate (adjusted to pH 4.0 with formic acid) at a flow rate of 0.5 mL min^−1^ The method was validated and shown to be sensitive, accurate (recovery values 76.16–92.57%) and precise (RSD < 10% for all compounds) with a linear range over several orders of magnitude. The method was successfully applied to rat serum and shown to be indicative of the endogenous levels of biomarkers within the rat body. The analysis of the biomarkers can provide insight into the allergic mechanisms associated with related diseases.

## 1. Introduction

Allergies can cause significant morbidity in children and several studies have revealed that their prevalence may be increasing, especially asthma [1,2,3,4]. The aetiology of allergy is complex, involving polygenic predisposition including major histocompatibility complex genes, dysregulation of T lymphocyte function and various environmental factors [2,3,4,5,6]. Allergic inflammation is the body’s natural physiological response to infection or injury, acting as a defense mechanism to remove and repair damaged tissue. The cardinal features of allergic inflammation are caused in large part by an increase in blood flow and vascular permeability and the ability for larger inflammatory mediators to cross the endothelial surface and travel and adhere to the site of the injury. As a result, current researches focus on developing quantitative analytical techniques to discover biomarkers of allergic inflammation that can be utilized to more accurately predict future disease risk.

**Figure 1:**
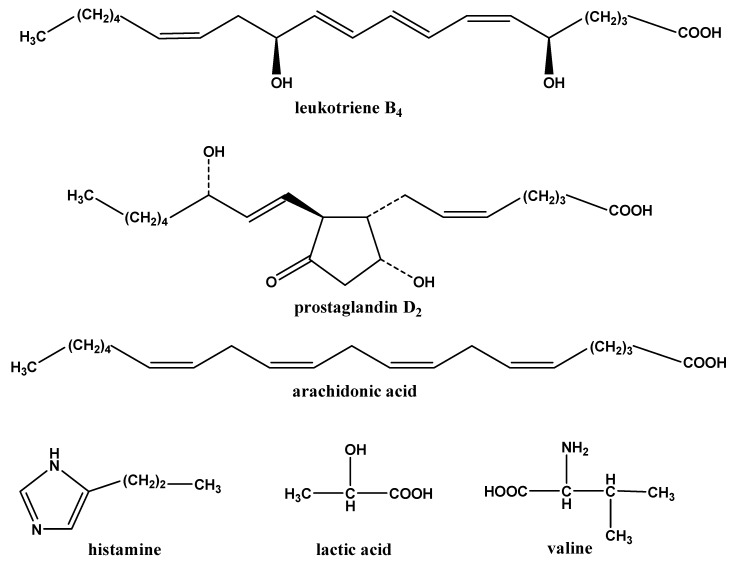
Structures of the compounds.

Current analytical techniques for the separation of biological matrices include high-performance liquid chromatography with both UV (LC-UV) [7,8,9,10] and fluorescence (LC-FL) [11,12,13,14] detection, capillary electrophoresis with UV detection (CE-UV) [15] and gas chromatography–mass spectrometry (GC–MS) [16,17,18]. The above techniques, which are popular methods for analyzing biosamples, have disadvantages that may limit their effectiveness in bioanalytical applications. UV detection requires active chromophores that many of the eicosanoids lack. In addition, LC-UV methods typically lack the sensitivity necessary to quantitate small levels of endogenous substances. Furthermore, methods that employ fluorescence or GC–MS analyses may sometimes involve complex and time-consuming sample purification and derivitization steps. LC coupled to mass spectrometry (either single or tandem, LC-MS or LC-MS/MS, respectively) is an increasingly popular choice for the analysis of biomarkers. The OVA-induced murine model is prevalent in the research of pathogenesis and therapeutics of allergic diseases [19,20,21]. In this paper, we established an OVA-induced allergy model with BN rats, and developed a RP-LC/MS method to separate and quantitate six different potential endogenous biomarkers (LTB_4_, PGD_2_, AA, HI, LA and VAL), which have physiological functions in allergic inflammation, from serum of allergic rats induced by ovalbumin (OVA) and normal rats, and compared the discrepancies between the model group and the control group. The structures of the compounds studied can be found in Figure 1.

## 2. Results and Discussion

### 2.1. Development of the LC-MS Method

To separate the six endogenous substances from other interferences in rat serum, various LC mobile phase systems were tested. Different combinations of mobile phases were tried using both isocratic and gradient elution. Finally, after several trials a gradient elution system containing 10 mM ammonium formate (adjusted to pH4 with formic acid) in water (A) and acetonitrile: formic acid (99.9:0.1, v/v) (B) was selected as the mobile phase. 

**Figure 2:**
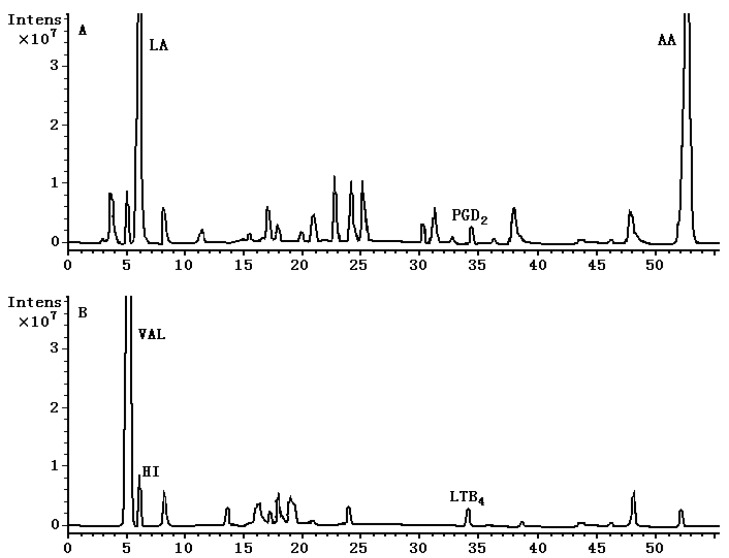
Representative total ion chromatograms (TIC) of the serum from the OVA-induced rat obtained in negative (A) and positive (B) ion electrospray. The retention time is 5.9 min, 34.5 min and 52.5 min for LA, PGD_2_ and AA respectively in negative ion mode, and the retention time is 5.3 min, 6.0 min and 34.1 min for VAL, HI and LTB_4_ respectively in positive ion mode.

Mass spectrometric conditions were optimized so as to achieve the maximum stable response of the parent ions and the major productions of the analyte fragments. Selective reaction monitoring afforded by tandem mass spectrometry has the great advantage of reducing interference and enhancing sensitivity over selected ion monitoring. The representative total ion chromatograms (TIC) obtained in negative and positive electrospray ionization mode are shown in Figure 2. 

The linearity of the standard curves was good over the entire concentration ranges of 1–50 ng mL^−1^ for LTB_4 _and PGD_2_, 1–50 μg mL^−1^ for AA and LA, 10–500 ng mL^−1^ for HI and 5–100 μg mL^−1^ for VAL as shown in Table 2. The correlation coefficients (*r*) ranged between 0.9973 and 0.9986. The LOD and LOQ for all six compounds, at a signal-to-noise ratio of 3 and 10, were as shown in Table 1.

**Table 1:** Standard curves, correlation coefficients (*r*) and linear ranges, LOD and LOQ of the six compounds in serum from rats (n = 3).

Compounds	Linear ranges	Linear equations	Correlation coefficients ( *r*)	LOD (ng mL^−1^)	LOQ (ng mL^−1^)
LTB4	1–50 ^a^	*y* = 0.5632*x* + 0.8554	0.9986	0.2	0.5
PGD2	1–50 ^a^	*y* = 4.8623*x* + 2.5414	0.9986	0.2	0.5
AA	1–50 ^b^	*y* = 0.8905*x* + 0.2334	0.9984	2.5	7.0
HI	10–500 ^a^	*y* = 0.6579*x* + 0.4889	0.9979	1.5	4.5
LA	1–50 ^b^	*y* = 1.1658*x* + 0.6996	0.9981	5.0	12.0
VAL	5–100 ^b^	*y* = 0.9825*x* + 0.7466	0.9973	2.0	8.0

^a^ ng mL^−1^; ^b^ μg mL^−1^

Accuracy and precision of the method were determined with quality control samples. The relative standard deviation (RSD) and mean values of the deviation from the amount added (% bias) were calculated. The results for serum are given in Table 2. The data showed good precision of the method with an intra- and inter-assay RSD of below 9%. The intra- and inter-assay accuracy as expressed by the bias ranged between 89.29% and 104.6% for the six compounds. 

The extraction recoveries of serum samples were also optimized in our preliminary studies by comparing protein precipitation reagents, such as MeOH, acetonitrile, EtOAc, liquid-liquid extraction by EtOAc and ethyl ether. The recoveries of the six compounds from serum were determined by spiked samples at three concentrations as shown in Table 2. The results showed that the recovery was 76.16%–92.57%.

The stability of the six compounds in serum was determined under various conditions according to the procedure described above. The results for serum are given in Table 2. No significant change of the six concentrations in serum was detected after exposing samples to three freeze–thaw cycles and recoveries were found to be 89.26%–112.5%.

**Table 2:** Intra- and inter-day precision, accuracy, extraction recoveries and stability of the six compounds in rat serum (n = 6).

		Intra-day				Inter-day				Recovery			Stability	
	spiked	Meansured	RSD	Accuracy		Meansured	RSD	Accuracy		Mean	RSD		Accuracy	RSD
Cpds			(%)	(%)			(%)	(%)		(%)	(%)		(%)	(%)
LBT_4 _^a^	5	5.05	1.42	101.00		4.95	1.33	98.96		81.08	3.60		97.50	5.41
	10	9.74	4.32	97.40		9.18	4.46	91.82		84.22	4.14		96.89	3.97
	40	36.87	6.55	92.18		39.40	7.17	98.49		85.72	4.08		94.31	4.16
PGD_2 _^a^	5	4.67	6.29	93.40		4.78	3.27	95.57		76.16	5.40		93.85	5.37
	10	9.81	1.92	98.10		8.93	5.36	89.29		79.33	3.92		97.14	1.35
	40	39.02	8.51	97.55		38.39	6.86	95.97		82.61	2.71		93.56	3.97
AA ^b^	5	4.78	1.40	95.60		4.69	6.86	93.85		81.46	2.47		101.7	4.09
	10	9.74	2.46	97.40		9.09	3.83	90.87		86.00	5.03		90.71	2.04
	40	36.55	5.98	91.38		38.81	6.51	97.02		85.05	3.08		91.19	3.76
HI ^a^	50	47.10	1.43	94.20		46.05	1.34	92.09		87.30	2.72		90.57	5.45
	100	98.02	6.73	98.02		90.72	2.93	90.72		83.92	4.77		95.57	4.49
	400	380.2	2.74	95.05		364.1	6.17	91.02		85.31	4.49		112.5	4.65
LA ^b^	5	5.23	5.87	104.6		4.66	8.45	93.14		92.57	5.68		93.09	1.96
	10	9.67	1.33	96.70		9.46	4.01	94.63		86.66	3.40		99.50	4.46
	40	36.49	2.24	91.23		36.64	1.19	91.59		83.24	0.73		95.87	2.87
VAL ^b^	10	9.83	1.27	98.30		9.82	0.35	98.19		84.33	3.59		89.26	0.48
	40	37.44	0.68	93.60		36.86	3.95	92.14		82.10	3.60		91.33	0.97
	80	78.46	4.21	98.08		77.54	1.91	96.92		83.70	1.70		96.58	2.90

^a^ ng mL^−1^; ^b^ μg mL^−1^

### 2.2. Application to Contents Analysis of Endogenous Substances in Serum

**Figure 3:**
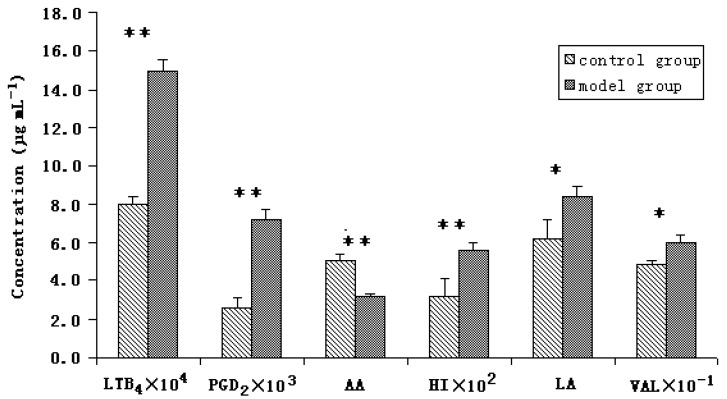
Concentration figure of the six endogenous substances in rat serum of the modal group and the control group. ** p < 0.01, * p < 0.05.

The method described here was successfully applied to compare the peak intensity of the six endogenous substances in serum of the two groups. The result showed that there were significant differences in contents of all the six endogenous substances between the control group and model group. LTB_4_ and PGD_2_ belonging to a family of proinflammatory lipid mediators, play an important role in the pathogenesis of allergic inflammation, the two endogenous metabolites are the biological oxidative metabolites of AA, responsible for a large variety of physiological functions including regulating inflammation. Compared with the control group, the concentrations of LTB_4_ and PGD_2 _in allergic rat serum were all increased, and the level of AA was decreased correspondingly. HI is one of the major mediators of the acute inflammatory and immediate hypersensitivity responses, its release could lead to the development of such symptoms as acute rhinitis, bronchoconstriction, cramping, diarrhoea or cutaneous weal and flare responses, therefore, the level of HI in serum of allergic rat was significantly higher than that in serum of normal rat, as shown in Figure 3. The concentrations of LA and VAL were also found increased in serum of modal group compared with the normal due to the metabolic disorder, which were also displayed in Figure 3. The statistical treatment of data was carried out using Microsoft Excel 2000.

## 3. Experimental

### 3.1. Chemicals, Reagents and Animals

LC-grade methanol and acetonitrile were purchased from Fisher Company (Fisher Scientific, USA). Formic acid of analytical grade was purchased from Tianjin Baoqi Tech. Reagent, Ltd., (Tianjin, China). LTB_4_, PGD_2_, AA, HI, LA and VAL were obtained from Sigma Co. (St. Louis, MO, USA). Triple-deionized water was purified by Milli-Q system (Millipore, Bedford, MA, USA). Male BN rats (180–200g), obtained from Wei Tong Li Hua Laboratory Animal Co. Ltd. (Beijing, China), were kept in a controlled-environment breeding room (temperature: 22 ± 1°C, humidity: 60 ± 5%, 12-h dark/light), with free access to common food and water in the first week. All experimental procedures were conducted in accordance with the European Community guidelines for the use of experimental animals and approved by the BeiJing University of Chinese Medicine Committee on Animal Care and Use.

### 3.2. Instrumentation

The LC used was an Agilent 1100 series LC (Agilent Technologies, Santa Clara, CA, USA) with a binary pump, on-line degasser, and a thermostated autosampler. The LC was coupled to an Agilent LC/MSD Trap XCT Plus mass spectrometer equipped with electrospray ionization (ESI) source. The data were obtained using LC/MSD Trap Software 5.3 (Agilent Technologies, USA).The separation was performed on a Prevail C_18_ column (250 × 4.6 mm I.D., 5 μm, Alltech Inc., USA).

### 3.3. Chromatographic Conditions

The injection volume was 10 μL. The column was maintained at 25 °C with a linear gradient elution of mobile phases A and B at a flow rate of 0.5 mL/min. Mobile phase A consisted of 10 mM ammonium formate (adjusted to pH 4 with formic acid) in water, while mobile phase B consisted of acetonitrile and 0.1% formic acid. Gradient elution was employed and was used as follows: started with 20% B and increased linearly to 40% B within 5 min, And then increased linearly to 60% B within 10 min, then increased linearly to 80% B within 20 min, at last increased linearly to 100% B within 25 min. Electrospray ionization (ESI) in the positive and negative ion mode was used as the ionization source. Nitrogen was used as the nebulizer gas and was maintained at a flow of 11.0 L min^−1^ with a nebulizer pressure of 35 psi. The gas temperature was set at 350 °C and the capillary voltage was 3000 V. The fragmentor voltage was set at 120 V and the gain was 2.0. The overall scan time was determined by 3 microscans and the maximum ion injection time as 8,000 ms with the automatic gain control (AGC) on. Mass spectra were recorded covering *m/z* 15–800 without any additional in-source fragmentation.

### 3.4. Sample Preparation

Twelve male BN rats were randomly divided into two groups, with each group containing six animals, and one group was regarded as the control group. The model group was established by intraperitoneally injecting 50 μg OVA, which was dissolved in 0.1 mL Al(OH)_3_ gel, for 14 days consecutively. The evaluation criterion of sensitization used to verify the allergic model was exhibited in Table 3. The control group was injected the same volume normal saline for 14 days. Blood were collected from the heart prior to sacrifice. Serum samples were then removed from the coagulated blood after centrifugation (10,000×*g*, 10 min, 5 °C), and were vortexed with three times acetonitrile for 30 s, then the mixture was centrifuged at 15,000×*g* for 10 min at 4 °C. The supernatant fluid was dried under a gentle stream of nitrogen gas. The residue was dissolved with acetonitrile and stored at −80 °C prior to LC-MS^2^ analysis.

**Table 3:** The evaluation criterion of sensitization.

Level	Result
-	Negative: normal
+	weakly positive: restlessness, piloerection, jitter, scratching nose
++	Positive: sneeze, cough, breathlessness, emiction, bowel, dacryorrhea
+++	strongly positive: dyspnea, wheezing rale, Peliosis, Instability of gait, jump, gasp, cramp, tropic, Cheyne-Stokes breathing
++++	very strongly positive: die

### 3.5. Calibration and Method Validation

Individual stock solutions of LTB_4_, PGD_2_, AA, HI, LA and VAL were prepared by dissolving samples in 1.0 mL methanol to create stock mass concentrations of 0.1 mg mL^−1^, respectively. Working standard solutions of all compounds were prepared by serial dilution in methanol from stock solutions to create the necessary concentrations. All solutions were stored at −80 °C until analysis. The calibration curves were constructed from three replicate measurements of six concentrations of each compound. The lower limit of detections (LODs) was determined during evaluation of the linear range of the calibration curve, and was defined as the lowest concentration level resulting in a signal-to-noise ratio of 3:1. The lower limit of quantifications (LOQs) was defined as the lowest concentration of the six compounds giving a signal-to-noise ratio of 10:1.

The intra- and inter-day precision, and accuracy of the six compounds were determined by quantitating six replicates a three concentrations on the same day and three consecutive days. Mean and relative standard deviations (RSDs) were calculated from validation samples values and used in the estimation of intra- and inter-day precision. Accuracy was assessed by comparison of the calculated mean concentrations to nominal concentrations. The recoveries of the compounds from serum were determined by spiked samples at three concentrations. The extraction recoveries were calculated by comparing peak areas extracted from rat serum with mean peak areas of the three same amounts of unextracted compounds prepared in MeOH solutions. Stability of the six compounds in rat serum was assessed for the freeze-thaw stability study, the samples were stored at −80 °C for 24 h, and then were left at room temperature for 1 h to thaw, the cycle was repeated three times and analysis was performed after the third cycle.

## 4. Conclusions

Previous researches have revealed that subtotal diseases are related to the disorders of amino acid and lipid metabolism. Discovery and quantitation of the potential biomarkers is one of the most important methods to gain insight into diseases. Chen [22] *et al*., studied the metabolic fingerprinting of urine in patients with liver cancer by LC/MS, revealing 21 endogenous metabolites as biomarkers in urine, which can provide assistance for disease diagnosis. Jie Zhang [23] *et al*., researched the serum metabolic characteristics in nephropathic patients and type 2 diabetes mellitus patients, and identified several potential biomarkers. 

AA and AA-derived eicosanoids are potent pro- and anti-inflammatory mediators, which play an important role in the pathogenesis of allergic inflammation. The detection of which may provide insight into the development of allergic inflammatory conditions. HI and VAL are two chemotaxins of allergic inflammation, and the interaction of histamine with the histamine H1 receptor mediates a variety of effects associated with symptoms of anaphylaxis and other allergic diseases [24]. High concentrations of LA are found under various pathophysiological conditions and are accompanied by an acidification of the environment. Establishing a sensitive and specific analytical method to detect the related endogenous substances conduce to penetrating investigate the pathogenesy and the course of allergy.

This method is the first reported method to use LC-MS to simultaneously analyze the main endogenous metabolites related with OVA-induced allergy which were compared between the model group and the control group. The method is linear over several orders of magnitude and sensitive enough to quantitate endogenous levels of important mediators. The present results demonstrated the significant discrepancies of the six potential endogenous biomarkers between OVA-induced rats and normal rats which are relative to allergic disease. Application of this method to rat serum can help aid in the development of improved therapeutic protocols for the treatment and prevention allergic diseases in clinical therapy.
